# One-pot neutron imaging of liquid–gas system: a parametric method study

**DOI:** 10.1007/s10967-025-10561-w

**Published:** 2025-11-18

**Authors:** Jonatan Šercl, Jongmin Lee, Eric Ricardo Carreón Ruiz, Martin Melčák, Jan Heyda, Ondřej Vopička, Pavel Trtik

**Affiliations:** 1 Department of Physical Chemistry, University of Chemistry and Technology, Technická 5, Prague 6, 166 28 Prague, Czech Republic; 2 PSI Center for Neutron and Muon Sciences, Paul Scherrer Institute, 5232 Villigen, Switzerland

**Keywords:** Neutron radiography, Liquid–gas interface, Petroleum, Natural gas, Surface tension, Diffusion

## Abstract

We present advancements in the one-pot neutron imaging method for investigating hydrocarbon-based liquid–gas interactions under varying pressure and temperature. Improvements in hardware enable the optimization of cell geometry and material, overall leading to reduced uncertainty in surface tension measurements. The integration of snubbers minimizes initial pressure surge, improving experimental stability. A Python-based pipeline is developed for image batch-processing, incorporating rigorous image reduction algorithms specific to the one-pot approach. This results in efficient data management and enhanced meniscus shape detection. Moreover, we provide a parametric study on chosen variables to further demonstrate the one-pot neutron imaging as a powerful tool for studying interfacial phenomena and fluid for a wide variety of applications.

**Supplementary Information:**

The online version contains supplementary material available at 10.1007/s10967-025-10561-w.

## Introduction

Petroleum and natural gas still remain the primary energy sources in the current infrastructure despite the growth of alternative fuels such as hydrogen. To ensure energy efficient and safe processing (e.g. freeze-out mitigation in natural gas liquefaction), it is necessary to understand the interactions between model liquids (benzene, toluene, ethylbenzene, xylene, methanol, and ethanol) and gaseous fuels (hydrogen, methane, and ethane) under varying pressure and temperature. The relevant industrial applications include natural gas liquids production, blending of natural gas with hydrogen, organic hydrogen carrier conversions, and geology of oil and gas reservoirs. Liquid–gas fuel interactions directly influence phase behavior, solubility, and potential for solid deposition in pipelines and processing equipment. Understanding surface phenomena at the liquid–gas interface is crucial for determining physical properties of fuel mixtures at the process conditions. As much as these process conditions can be difficult to be attained experimentally, they can be reached by means of molecular dynamics simulations, which, however, necessitate the provision of robust experimental data on the systems as calibration. Conventional single-purpose techniques, including pendant drop tensiometer, optical/X-ray imaging and spectroscopy methods, face limitations under high-pressure conditions and often lack sensitivity for liquid/gaseous fuels.

**Fig. 1:**
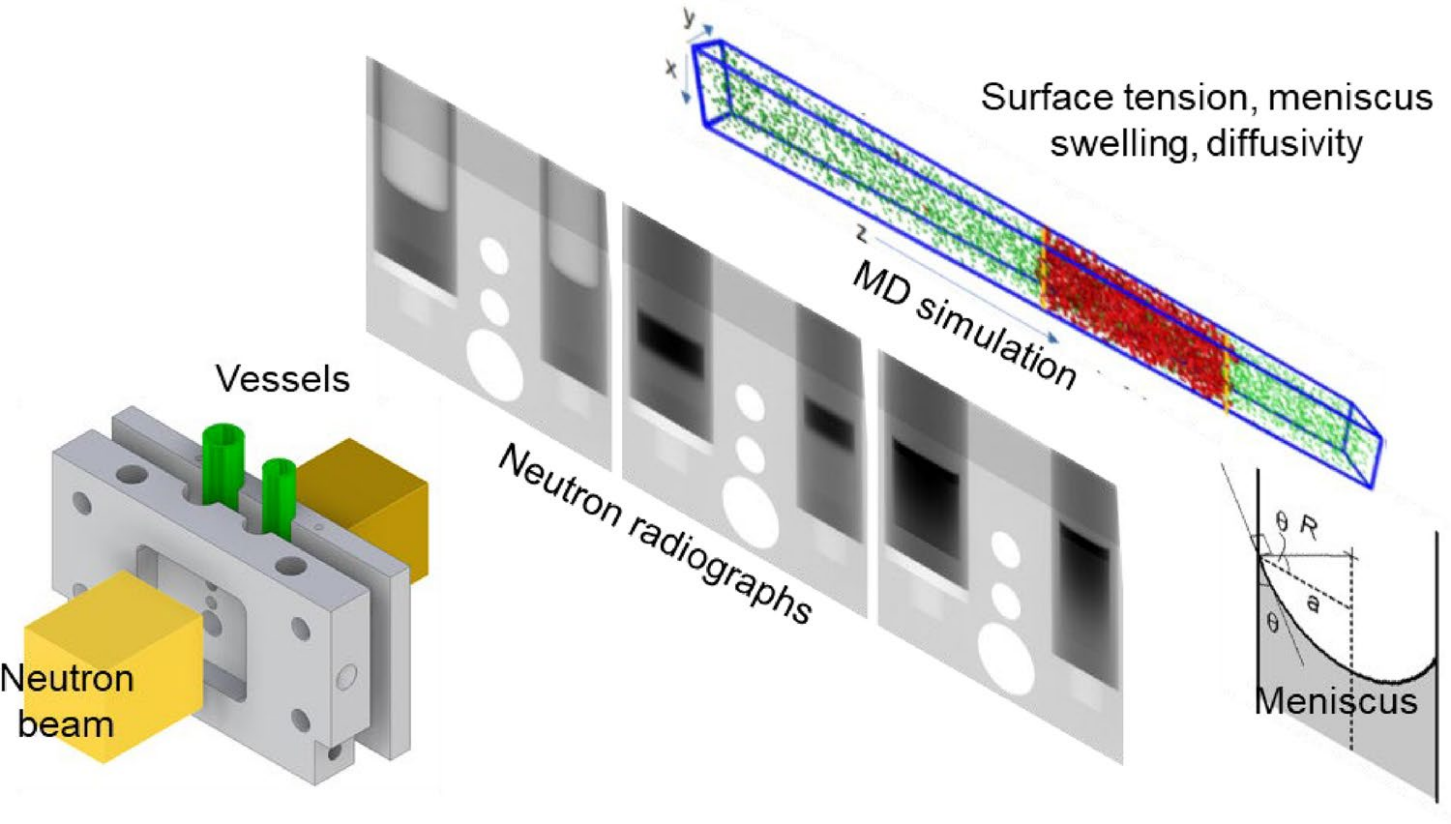
Schematic of one-pot neutron imaging of liquid–gas mixture in pressurized cells, MD abbreviates molecular dynamics

We pioneered the application of neutron imaging to study these liquid–gas interactions in situ, enabling a comprehensive analysis of physical properties under various conditions [1–3]. Neutrons can easily penetrate several centimeters of aluminum/titanium, while being highly sensitive to hydrogen-containing substances. Therefore, neutron imaging provides a high spatial and temporal information on liquid–gas interaction in a pressurized metallic cell, described as *one-pot neutron imaging method*. As seen in Fig. 1, multiple phenomena and the related physical properties can be studied. These include interface shape (surface tension), liquid level rise—swelling (apparent gas volume), and time-resolved distribution of the neutron attenuation in the liquid (solubility and dissolved gas diffusion).

In this work, we highlight recent advancements achieved in the 2nd generation of the *one-pot neutron imaging method*. Beyond improvements in the test setup and data handling, we conducted parametric studies on cylindrical cell materials and dimensions to evaluate their impact on the measured physical properties. By demonstrating the robustness of this method, this work aims to introduce it to a broader audience across various disciplines, with potential applications in oil and gas production and processing, CO_2_ capture and storage, hydrogen storage and transportation, and studies of colloidal systems.

## Experimental

### Apparatus

**Fig. 2:**
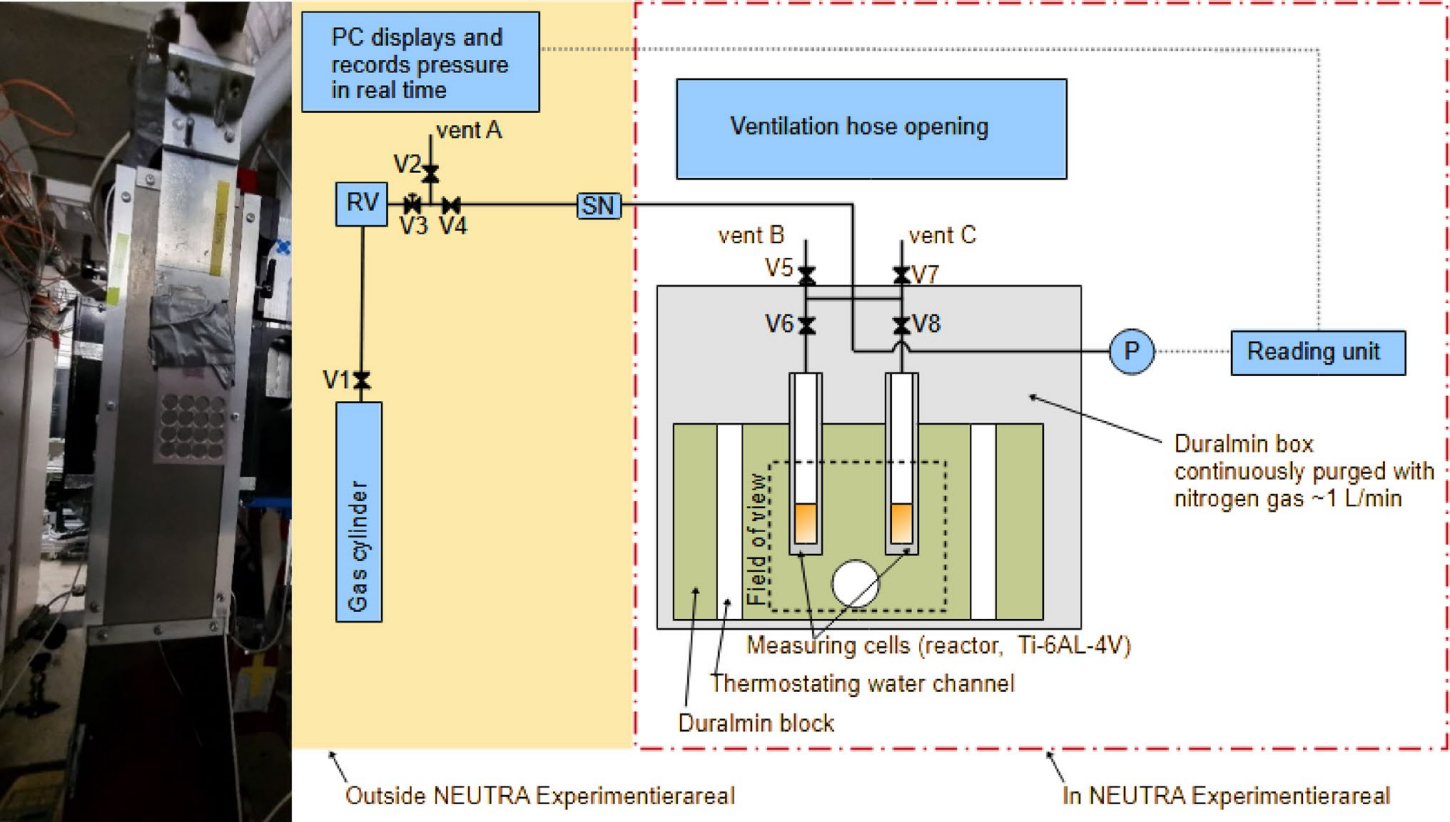
Photo (left) and schematic (right) of the setup at the beamline. The acronyms are as follows: personal computer (PC), reduction valve (RV), snubbers (SN), and pressure transducer (P) connected to a reading unit with data transmission to PC

The apparatus (Fig. 2) consisted of two cylindrical titanium alloy (Ti-6Al-4 V) cells with inner diameters of 9.0 mm and 12.0 mm (wall thickness of 1.5 mm), stainless steel (SS316) cells were studied for comparison. The roughness of the cell internal surface is critical for determining surface tension as it imposes changes of the contact angle and thus interface shape. The residual influence of the surface material and its roughness on the surface tension that is the property of the liquid–gas interface only (see below) needs to be studied. Therefore, cylindrical titanium alloy was machined by high precision computer numerical control (CNC) with a tolerance of 25 µm. The two cells were enclosed within a pair of duralumin blocks (Fig. 1). Duralumin (EN AW 6060) was used as it shows high thermal conductivity, mechanical stability, and neutron transmittance. The block was maintained at a constant temperature by circulating a thermal fluid through the engraved channels by a thermostat (Julabo, CD-600F). The temperature in the duralumin block was measured using a Pt100 thermometer (Greisinger, GMH 3710) placed in the duralumin block close to the cells and shielded against neutrons. Considering high thermal conductivities and precision machining, the temperature difference between measured (duralumin) and actual (cell) values can be neglected. The setup was enclosed in a duralumin box continuously purged with nitrogen gas to prevent moisture condensation and to mitigate the risk of creating an explosive atmosphere when working with pressurized flammable gases. The measurement cells were connected to the gas cylinder through all-stainless steel tubing. Five snubbers in series (Swagelok, SS-4-SA-EG) were connected into the gas inlet to dampen the initial pressure surge which deforms the meniscus temporarily due to gas convection. Pressure was monitored and recorded throughout experiments (Omega, PXM409-100BAV) to ensure accuracy and reproducibility. The measurement box containing the cells was installed on the sample stage of the beamline and aligned to the neutron beam (pitch) and gravity (roll).

### Experimental procedure

The cells (titanium alloy, stainless steel) were initially rinsed with acetone, evacuated using a vacuum pump (DUO 1.6 with oil mist eliminator, Pfeiffer), and flushed with nitrogen gas (N₂) to remove residual contaminants. After acquiring neutron image of the empty cells as a reference, the studied liquid was introduced into the measurement cells, followed by vacuuming to remove any remaining gas. This was achieved by manipulating the system valves to retain the liquid within the designated tubes and cells. Once the liquid-filled cells were imaged under vacuum (with the gas phase containing only the vapor), the system was pressurized to 1 bar with the measurement gas and allowed to reach equilibrium. After equilibration, the gas pressure was increased in a single step to the target value, and neutron images captured the dynamic evolution of the system.

### Neutron imaging and processing

Neutron imaging was performed at the NEUtron Transmission RAdiography (NEUTRA) beamline [4] of the Swiss Spallation Neutron Source (SINQ). Based on a 40-µm thick Gadox scintillator and a scientific complementary metal oxide semiconductor (sCMOS) camera (Hamamatsu ORCA Flash 4.0), a pixel resolution of 21.5 ± 0.5 µm was achieved with 30 s per frame. The details of the imaging setup have been described in our previous works [1].

In our previous works [1–3] (the 1st generation of the method), images were processed using multiple platforms, including ImageJ [5], Kiptool [6], and Python, requiring significant effort and time for data handling. For the 2nd generation, we developed in-house Python packages, implemented on Jupyter Notebook, to process neutron radiographs of in-situ cells containing liquid–gas mixtures under pressure. The processing pipeline consists of three steps: (1) file and folder organization, including the removal of low-intensity frames; (2) two-dimensional image processing including filtering, dark current/open beam corrections, registration, dose correction, alignment, scattered background correction [7, 8], referencing by dried cells, and cropping of individual cells; and (3) onion-peeling step to account for cylinder geometry [9]. The onion-peeling algorithm was used to reconstruct an axially symmetric radiography image into a single vertical tomographic slice at the sample center. The reconstruction is used to enhance the meniscus detection near the cell walls, which has a substantial effect on the surface tension calculation. The final image is further analyzed, and the physical materials properties are derived using our Maple script, as elaborated in the next section.

### Surface tension and solubility calculation

In-house developed Maple scripts yielded the calculation of diffusivity in the liquid phase, Henry’s law constant, apparent molar volume, and surface tension. The mathematical framework underlying these calculations is detailed in our previous study [3]. The calculations follow the import of the tomographic reconstruction imported as a text-format image (*.txt*) file.

The processing workflow in Maple consisted of three steps. In the first step, surface tension was determined from a single image acquired at 1 bar or vacuum (vapor pressure) by fitting the numerical solution of the Young–Laplace equation for the axially symmetric meniscus in gravity to the experimental meniscus shape. The Young–Laplace equation has the form [3, 10]

1 $$\frac{z \Delta \rho g}{\gamma } = \frac{{z^{\prime\prime}}}{{\left( {1 + (z{\prime} )^{2} } \right)^{3/2} }} + \frac{{z^{\prime}}}{{x\left( {1 + (z{\prime} )^{2} } \right)^{1/2} }}$$

where *z* denotes the height (shape) of the meniscus, *x* (ranging from 0 to *r*) is spatial coordinate with 0 meaning the tube center and *r* its inner diameter, *z′* and *z″* are derivatives with respect to *x*, $\Delta \rho$ is the difference of densities of the liquid and gaseous phases, *g* is gravitational acceleration, and *γ* stands for the surface tension. Equation (1) was solved for the conditions *z′*(*x* = 0) = 0 and *z′*(*x* = *r*) = cot(*θ*), where *θ* is the contact angle that is influenced by the cell material and its roughness. The second and third steps were iterative. In the second step, the shapes of the phase interfaces for the pressurized system were identified and fitted by the Young–Laplace equation, providing input (coordinates of the meniscus) for the third step. Then, diffusivity in the liquid was calculated by fitting the Fick’s second law to the observed temporal concentration distributions. The third step also provided concentration at the interface and thus Henry’s law constant, and processing of the liquid swelling (liquid level rise due to gas absorption) provided the apparent molar volume of the dissolved gas. Thus, the density distribution in the liquid phase was computed and used in the refined surface tension calculations. The used methods and models were summarized in the earlier study [3].

## Results and discussion

### Improvements in hardware

The design and machining tolerance of the cells and housing influence the accuracy and precision of physical properties derived from the tomographic reconstructions. The chosen materials must be neutron-transparent and leak-tight under high pressure (up to 120 bar). In our previous studies [1–3], a bench drill press with flat-bottom drill bits were used to machine titanium alloy cells, resulting in inconsistencies in wall thicknesses and inner diameters (of up to few hundreds of micrometers—see Fig. S1). Several cell designs were considered, including high-precision tubing with a welded bottom or a cap fitting. However, due to the requirements for high pressure and axial symmetry, we chose to use computer numerical control (CNC) machining on a solid Ti-6Al-4 V or SS316 rods with carbide drill bits. The duralumin block (as shown in Fig. 1) was also CNC machined. The typical CNC machining tolerance for the drilled hole diameter is in the order of few tens of micrometers. Within our imaging pixel resolution of 21.5 ± 0.5 µm, no significant variations in inner diameter were observed. The following sub-sections discuss the impact of specific parameters on the results.

#### Effect of cell diameter

The inner cell diameter significantly influences the sensitivity of the meniscus shape to surface tension (*γ*), for which the deformation of the interface by gravity is essential [3]. The sensitivity increases with the increasing cell diameter, reducing the uncertainty of surface tension when fitting the model, Eq. (1), to the experimental data. Additionally, large cells are expected to maintain a comparable absolute uncertainty in meniscus detection while providing a larger meniscus, which further enhances measurement accuracy [11]. However, increasing the cell diameter results in greater neutron attenuation through a liquid–gas mixture. This leads to decreased sensitivity for other parameters, such as diffusivity. In our new hardware, two cells in different sizes (inner diameters of 9 and 12 mm) were measured within a single experiment, where identical pressure and temperature were applied to both. Consistent with the assumptions above, the 12 mm diameter cells exhibited narrower 95% confidence intervals (CIs) for surface tension values as calculated using the Bonferroni method [12] (Table 1). This method accounts for the influence of the model deviation from the experimental data and the model sensitivity on its parameters.

**Table 1:** Dependence of the relative value of 95% CI (averaged) of the surface tension of *p*-xylene—methane system at different temperatures (293 K, 303 K, 313 K) calculated by Bonferroni method on the material and inner diameter of the cell

Material		Ti-6Al-4 V		SS316	
Inner diameter [mm]		12	9	12	9
Relative 95% confidence interval (Bonferoni method) of surface tension	1 bar	2.20%	3.47%	4.00%	8.58%
	100 bar	3.04%	4.06%	5.87%	10.79%

#### Effect of cell materials

We investigated the influence of the cell material on the measured surface tension. In our previous studies [1–3], the experimental cells were manufactured from titanium alloy (Ti-6Al-4 V). For comparison, stainless steel 316 was selected, as both materials are widely used in industrial applications due to their favorable mechanical properties and corrosion resistance [13].

Surface tension of *p*-xylene–methane mixtures was measured across a range of temperatures (20, 30, and 40 °C) and pressures (1 and 100 bar). The results demonstrated that Ti-6Al-4 V cells provided lower 95% CI for surface tension (Table 1). Furthermore, measurements conducted in Ti-6Al-4 V cells showed reduced sensitivity to variations in cell radius compared to SS316 (Table 1). This improved performance may be attributed to the lower surface energy of Ti-6Al-4 V relative to SS316 [14]. In addition, titanium alloys exhibit lower bacterial adhesion compared to stainless steel [15], which enhances their suitability for long-term experimental use. Based on these advantages, Ti-6Al-4 V was selected as the standard material for all subsequent experiments.

#### Alignment due to gravity and beam

The cells were aligned to gravity and beam by tracking three through holes in the duralumin block between the cells. Orientation relative to gravity has a substantial influence also for the other methods of surface tension measurement [16]. In this work, the effect of cell pitch on surface tension measurements was investigated within a ± 1° range motor on the sample stage. This analysis was conducted with perdeuterated liquid *p*-xylene in the absence of other gas (gas phase consisted of the *p*-xylene vapor) across a temperature range of 0 °C to 30 °C, since surface tension decreases with increasing temperature. The results (Table 2) showed no significant variation in surface tension due to tilt, with all deviations falling within the average uncertainty range determined by the Bonferroni method. These findings confirm that minor tilting does not influence the accuracy of surface tension measurements, thus showing the robustness of the method.

**Table 2:** Comparison of the average deviation caused by pitch within a ± 1° range with the average deviation computed using the Bonferroni method for each measurement at different temperatures, cell from titanium, inner cell diameter 9 mm

Temperature [°C]	Average surface tension [mN/m]	Average 95% CI (Bonf. method) [mN/m]	Average deviation with pitch [mN/m]
0.0	28.60	0.74	0.33
7.0	27.95	0.75	0.56
13.0	27.82	0.85	0.48
20.0	26.84	0.73	0.59
30.0	26.42	0.81	0.33

#### Improving gas flow characteristics

One of the deficiencies in the 1st generation of the *one-pot neutron imaging method* was the quick (manually controlled) pressurization. The pressure surge can induce unintended mixing of the gas and liquid phases, potentially affecting the accuracy of diffusivity and apparent molar volume determination. However, due to the rapid nature of this phenomenon, its precise impact could not be investigated otherwise than by repeating the experiments with different levels of liquids [1]. To mitigate this issue, snubbers were installed on the pipeline to attenuate the surge, thereby minimizing its influence on the measurement. This modification also improved the repeatability of the pressurization process, leading to more consistent experimental conditions. Furthermore, pressure gauge reading with data logging has been installed in the 2nd device generation. The pressure change during the pressurization was real-time monitored, providing a better understanding of the pressure increase dynamics and its potential impact on the measurements. Furthermore, the recorded data facilitated more precise control of the experimental conditions, enhancing the overall reliability and reproducibility of the results.

During the pressurization, the dependence of pressure on time exhibited a sigmoidal shape, which was well described by the equation:

2 $$p = A \cdot (1 - e^{ - ct} - c \cdot t \cdot e^{ - ct} )$$

where *p* represents pressure, *A* is the maximum pressure reached minus the starting pressure (vacuum or 1 bar in this work), *t* is time, and *c* is reciprocal time constant that characterizes the rate of pressurization. A lower value of *c* (higher 1/*c*) corresponds to a slower pressurization process. The use of Eq. (2) allowed for better control of the experimental conditions*.*

The time constants (1/*c*) in Eq. (2) obtained from a series of pressurization measurements are presented in Table 3. The data indicate that the addition of five snubbers significantly slows down the pressurization, effectively minimizing the surge. Notably, the effect of opening the pressure valve on the gas cylinder appears to be almost negligible, suggesting that the system response is primarily governed by the (invariant) characteristics of the snubbers and thus well reproducible.

**Table 3:** Time constants of the apparatus pressurization with methane from 1 bar to the indicated pressure

	Time constant (1/*c*) [s]		
Pressure [bar]	No snubbers	1 snubber	5 snubbers
100	0.69	1.67	2.37
50	0.70	1.72	2.40

### Improvement in image processing software

In our 2nd generation of the *one-pot neutron imaging method*, image processing is crucial for ensuring data integrity and reproducibility of results. While our previous works [1–3] introduced the foundational image processing framework, we have implemented additional algorithms to enhance data quality and coherence. This framework [17] used for this and other applications is open to the public. As noted above, all image processing steps are executed in Jupyter Notebook, based on Python packages originally developed for time-series radiograph datasets in hydrogen fuel cell applications. Moreover, metadata are embedded in image files to track input parameters and processing steps. The main features are summarized below.

#### Rigorous 3D filtering for white spot

When gamma rays, produced by neutron-matter interactions, hit the detector, pixel with extremely high values (white spots) are created in the image. To identify these white spots, a Gaussian curve is fitted to the intensity histogram of a selected area, preferably a low intensity region where gamma-induced spots stand out against background. A 99% confidence interval criteria is used as a threshold for white spot detection. Then, white spot values are replaced by using three-dimensional (3D) filter, accounting for neighboring pixels and adjacent images.

#### Low-intensity frame dropping

The PSI proton accelerator provides proton to multiple facilities in addition to SINQ, including the ultra cold neutron (UCN) source that operates in several seconds pulses applied in several minutes cycle. An UCN kick-in interrupts neutron beam production in SINQ, significantly reducing overall counts in affected images by up to 30%. Therefore, the identification of images acquired during the UCN kick-in was automated and the identified images removed from further analysis, as shown in Fig. S2.

#### Registration and alignment

Physical displacement of the cells contained in the Duralumin housing was unavoidable due to liquid/gas replacements as well as pressure and temperature changes recorded over several days of beamtime campaigns. To mitigate this, a movement tracking algorithm was implemented to track features in a selected region of a reference image and register subsequent images with sub-pixel precision. Figure S3 demonstrates the effectiveness of registration by tracking features in the through-holes in the reference (dry) image. This registration step significantly improves downstream processing, for example, enabling precise cropping of individual cell without manually defining edges (cell walls) towards the onion peeling step.

#### Scattered background matching

Scattered neutrons from both the sample and detector introduce false intensity values in the transmission image [7, 8]. In our previous works, the scattered background contribution was captured using black body (BB) dots superimposed on empty cell images. For further improvement, we evaluated and applied scattered background (SBKG) correction using cells with a liquid–gas mixture at varying pressure levels. Examples of BB images from the ambient pressure to 100 bar and corresponding SBKG images are shown in Fig. S4.

#### Open beam versus dry referenced

**Fig. 3:**
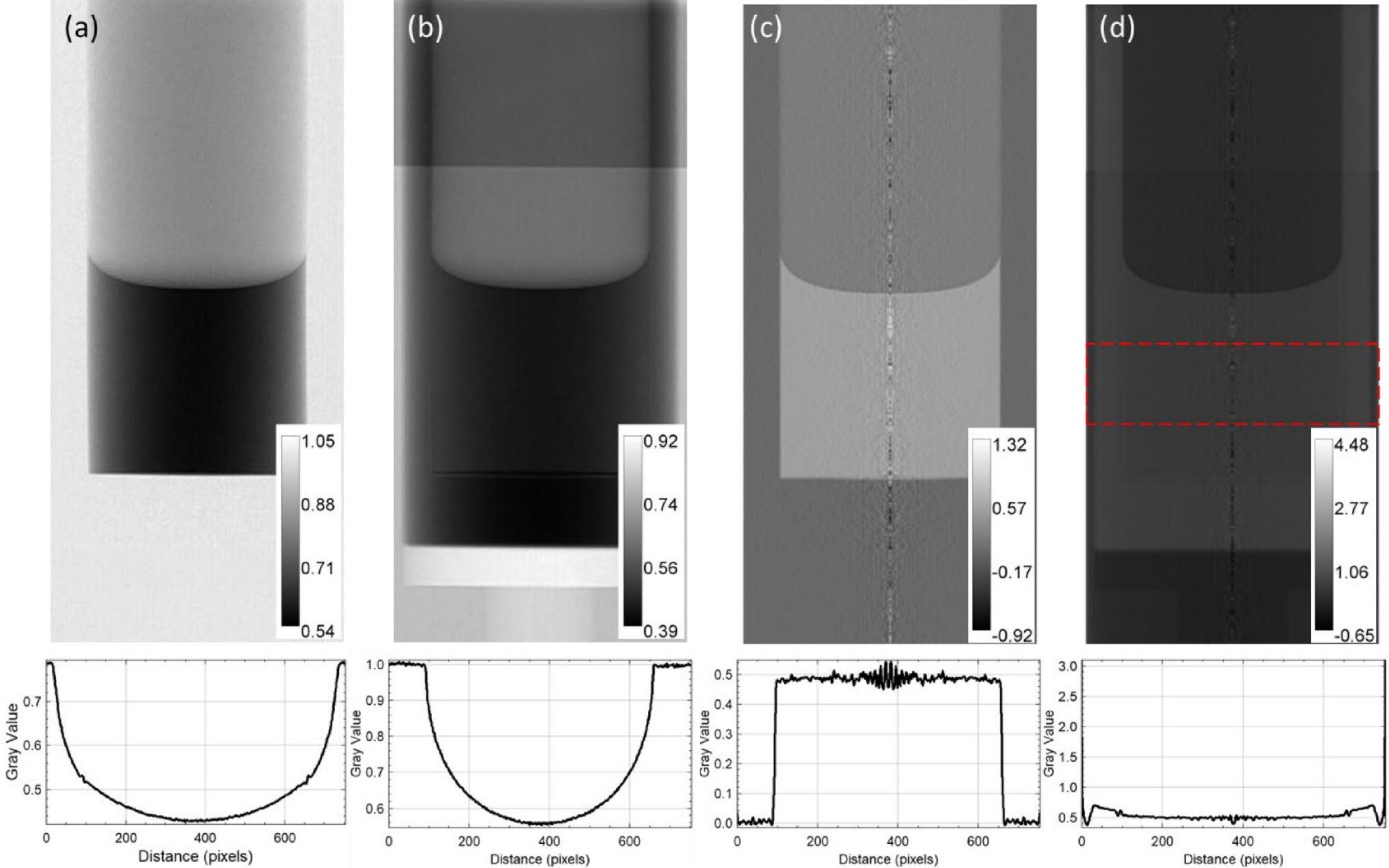
A 12 mm inner diameter cell containing hydrogen gas and methanol at 25 °C and 100 bar: **a** referenced by an empty cell image; **b** referenced by open beam image; **c** onion peeled image of empty cell referenced; and **d** onion peeled image of open beam referenced. In **a** and **b**, pixel values represent transmission level, whereas for **c** and **d** attenuation coefficients. Horizontal profiles are based on the region in the red dotted box in (d)

Previously (1st device generation), in-situ cell images were normalized using open beam, followed by onion peeling, as shown in Fig. 3b and d, respectively. This approach provides an intuitive representation, where pixel values in the final single-slice 3D image correspond to the attenuation coefficient. However, background structures, such as Duralumin block and housing, are not axially symmetric, which violates a key assumption in the onion peeling step. Therefore, in-situ cell images were normalized by empty cell images instead; only the liquid–gas mixture remains visible without contributions from the background, as shown in Fig. 3a and c. This background removal enhanced the detection of the meniscus and triple-phase interface among liquid, gas, and the cell inner wall.

#### Onion-peeling input parameters

The onion-peeling step requires two user inputs (offset and tilt) to align the rotational axis to the image center, similar to typical tomography reconstruction methods. In our previous work, these parameters were determined manually via a trial-and-error approach. With the implementation of the registration and alignment steps described above, the tilt and offset adjustments were no longer required. Nonetheless, we performed a parametric study of tilt and offset, as shown in Table S1. The surface tension values remained consistent for tilt values between − 1.0*°* and + 0.5*°* and offset values between − 0.4 and 0.2 pixels.

#### Improvement in meniscus detection

A critical initial step in the data processing workflow in the calculation of physical properties in Maple is the accurate detection of the meniscus shape and position. In earlier studies, the meniscus was manually traced, which was used as input for subsequent processing in Maple. In the new framework, an automated meniscus detection was implemented based on local maximum derivative in the intensity profile. This approach improved the reproducibility of the results by eliminating human error in the evaluation process.

## Conclusions

This study presents advancements in the one-pot neutron imaging method for investigating liquid–gas interactions under varying pressure and temperature. We have implemented hardware and software improvements to enhance data integrity and reproducibility. The use of high-precision CNC machined parts resulted in better wall thickness control of cells, while titanium alloy cells exhibited lower uncertainty in surface tension measurements compared to stainless steel. The addition of snubbers repeatedly reduced pressure surges and mitigated the uncontrolled mixing of liquid and gas.

We migrated existing image processing workflow to Python-based pipeline. Key improvements include robust white spot filtering, image registration, low-intensity frame removal, and advanced SBKG corrections. In Maple, automated meniscus detection was implemented based on local derivative maxima to reduce user dependence.

While these advancements define the 2nd generation of the reinforced *one-pot neutron imaging method* being a quantitative analysis tool providing multiple system characteristics from one experimental run, further efforts will focus on spectroscopic neutron imaging [18] to enhance the analysis of liquid–gas interaction. This technique enables in situ observations of dynamic processes in protonated species without the need of deuterated materials [19]. This will broaden the applicability of this technique by mitigating constraints related to deuterated materials availability and costs.

## Supplementary Information

Below is the link to the electronic supplementary material.

Supplementary file1 (DOCX 1740 kb)
