# Supplementary material for: One-pot neutron imaging of liquid–gas system: a parametric method study
Source: J Radioanal Nucl Chem. 2025 Nov 18;334(12):8921–8. doi: 10.1007/s10967-025-10561-w (PMC12827288; doi:10.1007/s10967-025-10561-w)
Supplement: Supplementary file 1 — Supplementary file1 (DOCX 1740 kb) [file 10967_2025_10561_MOESM1_ESM.docx]

Supplementary information

**
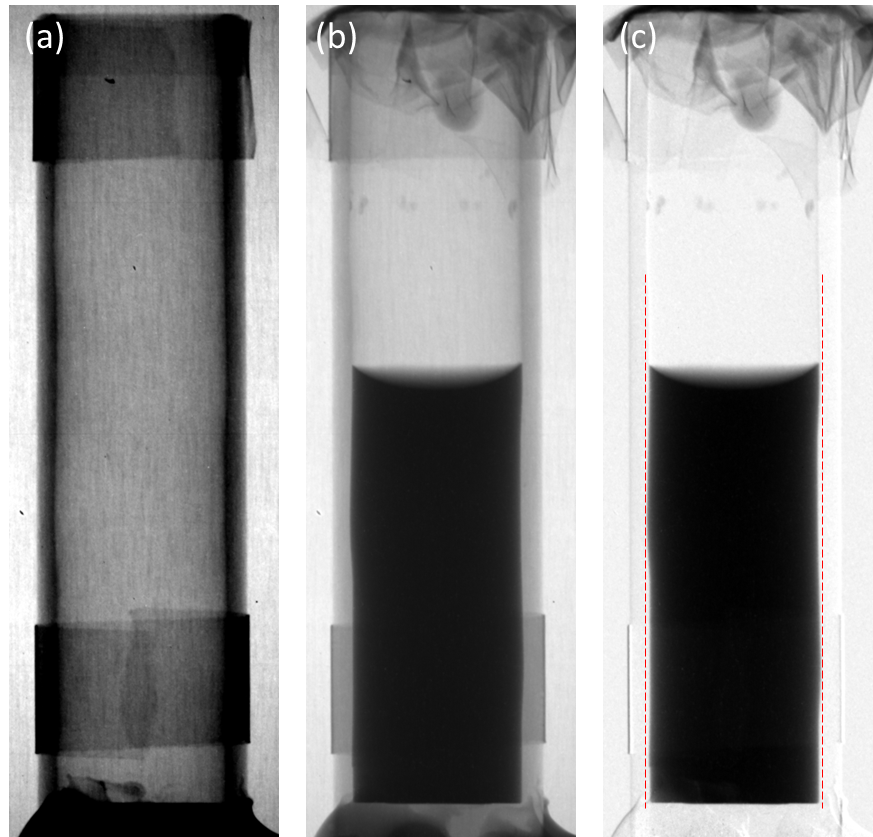
**

**Fig. S1.** Neutron radiograph of a 12 mm inner diameter cell used in the previous studies. (a) transmission images of an empty cell, (b) cell with water referenced by open beam, and (c) cell with water referenced by empty cell. The inner diameter varied in the range of several hundred micrometers. In new cells (CNC machined), the inner diameter variation was not observed in neutron images, indicating consistency within the spatial resolution, ± 50 µm.


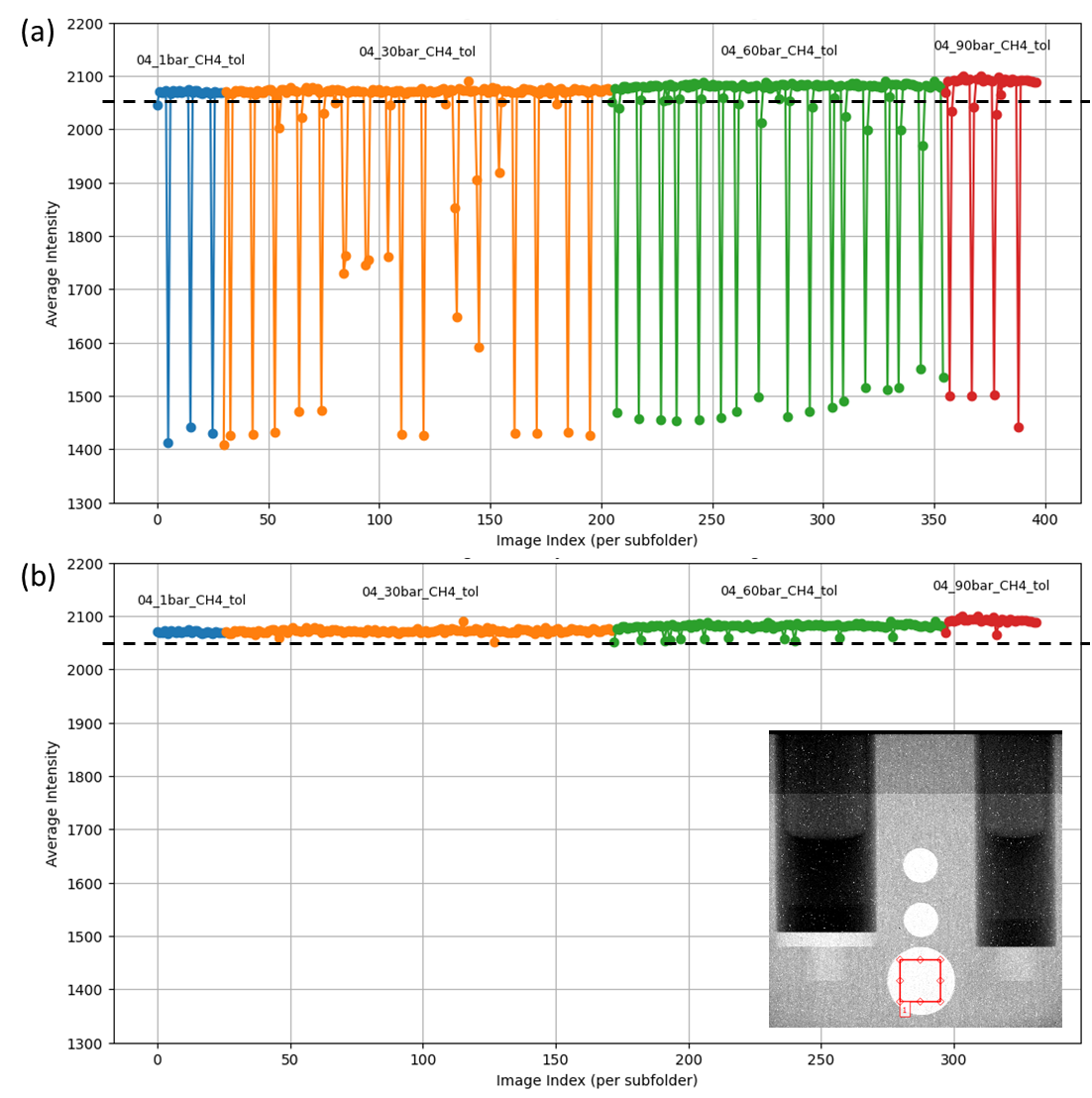


**Fig. S2.** Average pixel values for a selected region over 390 consecutive images: (a) affected by UCN operation showing intensity drop, and (b) after low intensity frame dropping with a cut-off threshold of 2050. Each color represents varying pressures from 1 to 90 bar with methane gas over toluene.


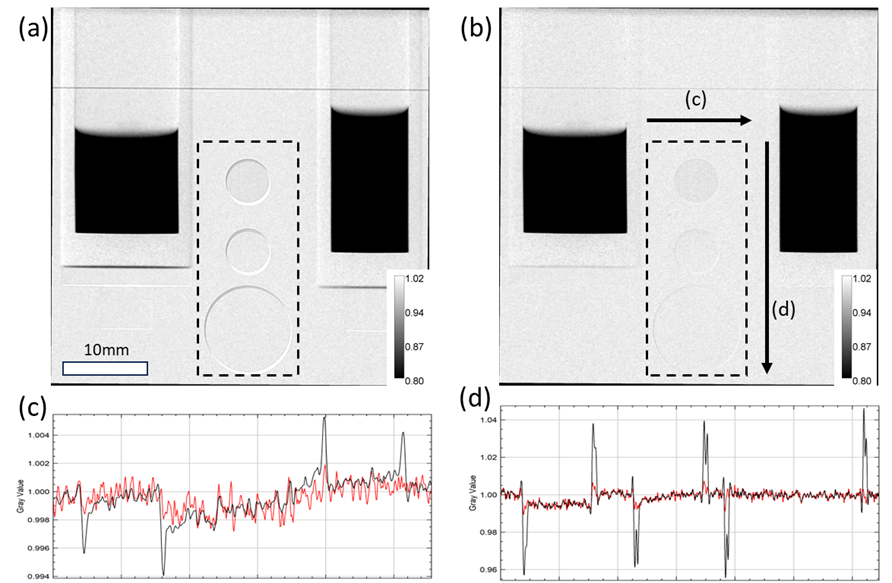


**Fig. S3**. Dry referenced images without (a) and with (b) registration. The region in the dotted box was used as tracking features. (c) and (d) represent the horizontal and vertical profiles of the selected area, respectively, before (black) and after registration (red).


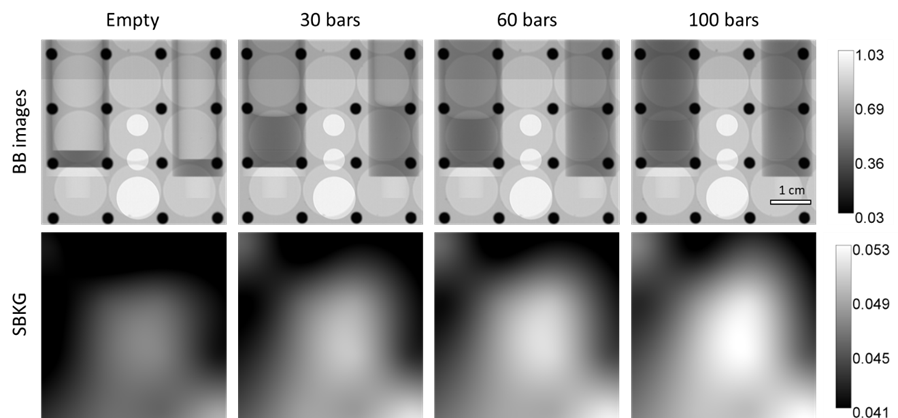


**Fig. S4.** Images of a black body (BB) grid placed in front of empty cells and cells filled with methane and methanol at 30, 60, and 100 bar and 40 °C. (top) The corresponding scattering background (SBKG) images showing increasing SBKG with pressure.

**Table S1**. Parametric study of offset and tilt during the onion-peeling step. Surface tension [mN/m] and average 95 % CI (Bonferroni method) [mN/m] are computed for offset values between -4 and +4 pixels and tilt values between -0.8º and +0.8º (*p*-xylene-methane mixture). The green, yellow, and orange regions indicate with high to low confidence levels, respectively.

|  |  |  | **Offset [pixels]** | | | | | | | | | | | | | |
| --- | --- | --- | --- | --- | --- | --- | --- | --- | --- | --- | --- | --- | --- | --- | --- | --- |
|  |  |  | -4 | -3 | -2 | -1 | -0.5 | -0.25 | 0 | 0.25 | 0.5 | 1 | 2 | 3 | 4 |  |
| **Tilt [degrees]** | -0.8 | Surf T. |  |  |  |  |  |  | 0.031 |  |  |  |  |  |  |  |
|  |  | Bonf |  |  |  |  |  |  | 0.002 |  |  |  |  |  |  |  |
|  | -0.6 | Surf T. |  |  |  |  |  |  | 0.029 |  |  |  |  |  |  |  |
|  |  | Bonf |  |  |  |  |  |  | 0.001 |  |  |  |  |  |  |  |
|  | -0.4 | Surf T. |  |  |  | 0.028 |  |  | 0.028 |  |  |  |  |  |  |  |
|  |  | Bonf |  |  |  | 0.001 |  |  | 0.001 |  |  |  |  |  |  |  |
|  | -0.2 | Surf T. |  |  |  |  |  | 0.027 | 0.027 | 0.027 |  |  |  |  |  |  |
|  |  | Bonf |  |  |  |  |  | 0.001 | 0.001 | 0.001 |  |  |  |  |  |  |
|  | 0 | Surf T. | 0.034 | 0.030 | 0.028 | 0.027 | 0.027 | 0.027 | 0.027 | 0.027 | 0.027 | 0.027 | 0.027 | 0.028 | 0.032 |  |
|  |  | Bonf | 0.003 | 0.002 | 0.001 | 0.001 | 0.001 | 0.001 | 0.001 | 0.001 | 0.001 | 0.001 | 0.002 | 0.002 | 0.003 |  |
|  | 0.2 | Surf T. |  |  |  |  |  | 0.027 | 0.027 | 0.027 |  |  |  |  |  |  |
|  |  | Bonf |  |  |  |  |  | 0.001 | 0.001 | 0.001 |  |  |  |  |  |  |
|  | 0.4 | Surf T. |  |  |  |  |  |  | 0.025 |  |  |  |  |  |  |  |
|  |  | Bonf |  |  |  |  |  |  | 0.002 |  |  |  |  |  |  |  |
|  | 0.6 | Surf T. |  |  |  |  |  |  | 0.024 |  |  |  |  |  |  |  |
|  |  | Bonf |  |  |  |  |  |  | 0.003 |  |  |  |  |  |  |  |
|  | 0.8 | Surf T. |  |  |  |  |  |  | 0.024 |  |  |  |  |  |  |  |
|  |  | Bonf |  |  |  |  |  |  | 0.004 |  |  |  |  |  |  |  |
